# Shockingly Unequal: Addressing Sex-Specific Needs in Cardiogenic Shock

**DOI:** 10.1016/j.jscai.2025.103714

**Published:** 2025-05-20

**Authors:** Michele L. Esposito, Susanna Price, Jacqueline Tamis-Holland

**Affiliations:** a Division of Cardiology, Department of Medicine, Medical University of South Carolina, Charleston, South Carolina; b Cardiology and Critical Care, Royal Brompton and Harefield Hospitals, Guy’s and St Thomas’ NHS Foundation Trust, London, United Kingdom; c National Heart and Lung Institute, Imperial College, London, United Kingdom; d Cleveland Clinic, Heart, Vascular and Thoracic Institute, Cleveland, Ohio

While advancements in the management and treatment paradigms for cardiogenic shock (CS) have evolved significantly over the past 2 decades, mortality rates remain high, approaching 40% in some cohorts,^1^ with notably higher mortality in women than that in men.^2^ In this issue of *JSCAI*, Baron et al^3^ provide an expert consensus statement on the management of CS in women with evidence-based guidance and gaps for consideration when caring for women with CS.^3^ This comprehensive document affords the practicing clinician with key points and useful information and allows for a greater understanding of sex-specific needs in CS.

The authors begin by emphasizing the lack of evidence-based therapies for women with CS. Historically, women have been underrepresented in clinical trials, which makes conclusions drawn from these data sets difficult to extrapolate for the general population. This discrepancy in clinical data leads to the risk of selection bias, where treatment protocols and recommendations may not accurately reflect the unique needs of female patients. It is crucial to remain mindful of the data sets being used (ie, clinical trial data, unselected population of patients, or registry data) to evaluate treatment options and differences in outcomes between sexes. Are we relying on selected data that may not be fully representative of the broader population or are we considering data that truly reflects the complexities of treating women with CS?

In their review of the literature, the authors note the clinical presentation of acute myocardial infarction (AMI) differs by sex. Women are older than men, have more comorbidities, and present with greater levels of hemodynamic compromise. Women with AMI-CS are more likely to experience delays in care, including longer times to percutaneous coronary intervention (PCI) and a higher likelihood of being referred to non-PCI centers. Additionally, women with AMI-CS are more likely to present with non–ST-elevation myocardial infarction (non-STEMI) rather than STEMI, which can delay referrals for invasive angiography. Accordingly, the authors recommend that it is imperative for health care institutions to examine and address local delays in care to reduce time to PCI for women and to consider early coronary angiography and revascularization in women presenting with AMI. This is further emphasized by recent ACC-AHA guidelines for managing acute coronary syndromes, which recommend an early invasive approach for all intermediate to high-risk patients with non-STEMI (regardless of sex).^4^

Aside from AMI-CS, notable sex differences exist in the presentation and management of heart failure-cardiogenic shock (HF-CS). The authors indicate that women who present with HF-CS, such as AMI-CS, tend to be older than men and have more comorbidities. Women are less likely to undergo pulmonary artery catheter monitoring, to receive temporary mechanical circulatory support (tMCS) or advanced surgical therapies. Knowledge of these disparities and a team-based approach may help ensure timely application of diagnostic and therapeutic options for women. Similarly, for sex-specific HF-CS, such as peripartum cardiomyopathy, a multidisciplinary approach is critical. The authors appropriately emphasize collaboration between cardiovascular and obstetric specialists, including maternal–fetal medicine, for optimizing care for these patients. They provide useful tips for vasopressor support, the safety and monitoring of various anticoagulants in pregnancy, careful patient positioning (to avoid aortocaval compression), and the importance of fetal monitoring. Decision-making regarding tMCS for this population of patients with HF-CS must be weighed against their presentation, comorbidities, and likelihood of clinical improvement.

The adoption of tMCS for CS is growing. Current guidelines provide IIa recommendation for microaxial flow pumps in the setting of STEMI-CS and another IIa recommendation for tMCS as a bridge to surgery in patients with mechanical complications of AMI^4^; however, much evidence supporting interventions in CS has been derived from studies with predominantly male enrollment. For example, in the DanGer Shock subgroup analysis, the relative mortality benefit derived from microaxial flow pumps compared with standard of care was not noted in women.^5^ Since DanGer Shock was underpowered to evaluate differences based on sex, these results must be contextualized within that limitation. Regardless of the potential benefit of early use of tMCS in CS, women are less likely to receive this intervention.^2^^,^^6^ This disparity is concerning because observational studies suggests that women may derive a greater survival benefit from early tMCS use, particularly pre-PCI in AMI-CS, as supported by data from the international cVAD registry.^7^^,^^8^ When focusing solely on the group of patients treated with tMCS, registry data have reported mixed results, with some studies demonstrating higher mortality in women,^9^ while others suggest similar survival to men.^7^^,^^8^^,^^10^ More data are needed to inform device selection and timing in female patients, especially in the form of randomized controlled trials.

The current consensus document emphasizes that bleeding and vascular complications, particularly those related to access sites, are more common in women than those in men in the setting of AMI-CS and with tMCS. These complications not only are more frequent but also have a greater impact on survival in female patients. Recent advancements, such as the development of smaller profile pumps, offer a potential alternative for improving vascular outcomes in women by reducing the risk of complications associated with large-bore arterial access. These innovations highlight the importance of tailored interventions and device development considerations to address the unique anatomical characteristics of women. Rather than limiting tMCS device use in women with CS, the authors emphasize mitigating risk by emphasizing safe vascular access techniques and management protocols for indwelling tMCS devices.

In summary, while significant progress has been made in the management and treatment options for CS, low enrollment of women in clinical trials of CS has resulted in evidence that is heavily male dominated. Within the constraints of the available data to guide care, it is critical that we remain cognizant of the unique presentations, complication profiles, and responses to treatment for women with CS. Moving toward a precision-based approach to care by addressing the unique challenges that women face in the management of CS, as outlined in the current consensus document, has the potential to improve outcomes in this critically ill cohort of patients.
